# THE COVID-19 PANDEMIC AND OBJECTIVE SOCIAL ISOLATION AMONG THE OLDER ADULTS IN THE UNITED STATES

**DOI:** 10.1093/geroni/igae098.2339

**Published:** 2024-12-31

**Authors:** Jiamin Yin, Christopher Seplaki

**Affiliations:** University of Rochester, Rochester, New York, United States; University of Rochester School of Medicine and Dentistry, Rochester, New York, United States

## Abstract

Experiences of social isolation are common among older adults and were amplified by the COVID-19 pandemic, particularly among those aged 80+. However, we do not know if such age differences in the experience of social isolation are due to the pandemic or if the underlying mechanisms driving social isolation may differ across age groups. We use a difference-in-difference design to compare the change in social isolation before and after 2020 across older adults aged 80+ versus those 65-79 years using data from the National Health and Aging Trends Study to constructed a composite measure of objective social isolation that includes four domains (living arrangement, communication regarding important matters, participation in religious and other activities). Our preliminary results suggest that adults aged 80+ years were found 3.7% (Standard Error (SE): 0.014) more likely than those aged 65-79 years to experience social isolation in 2020. Additional analysis of the four social isolation domains suggest that “older” older adults were 6.8% (SE: 0.016) more likely to report no participation in other activities than the “younger” older adults in 2020. In contrast, they were 3.5% (SE: 0.010) less likely than the “younger” older adults to live alone in 2020. No significant effect was observed in other domains. Our preliminary findings highlight the greater vulnerability of adults aged 80 years and the potential importance of developing interventions to address social isolation among this subgroup of vulnerable older adults.

